# Exploring age-related differences in metacognitive self-regulation: the influence of motivational factors in secondary school students

**DOI:** 10.3389/fpsyg.2024.1383118

**Published:** 2024-06-25

**Authors:** Ioannis Katsantonis

**Affiliations:** Psychology, Education and Learning Studies Research Group, Faculty of Education, University of Cambridge, Cambridge, United Kingdom

**Keywords:** age differences, achievement goals, metacognitive self-regulation, self-efficacy, measurement invariance, Cholesky decomposition

## Abstract

**Introduction:**

Metacognitive self-regulation is a crucial factor that promotes students’ learning and achievement. However, the evidence regarding age differences in metacognitive skills is rather mixed, with some evidence pointing toward further refinement and development and other evidence suggesting declining levels. Academic motivation, an important antecedent of metacognitive self-regulation, has also been reported to decline steeply in adolescence. Hence, this raises the question whether there are any age-related differences in academic motivation and metacognitive self-regulation of adolescents and whether age differences in academic motivation drive decreases in metacognitive self-regulation.

**Method:**

A large sample size of 1,027 Greek adolescents (ages 12–16, *M*_age_ = 13.95, *SD* = 0.78) was utilized in the present study. Multigroup measurement invariance analyses were deployed to compare the latent means of motivational factors (self-efficacy, task value, mastery, and performance goals) and metacognitive self-regulation across age groups. Cholesky decomposition was applied to test the independent contribution of motivational factors to and the indirect effects of age on metacognitive self-regulation.

**Results:**

Invariance analyses revealed scalar invariance for metacognitive self-regulation, language self-efficacy, mastery and performance goal orientations and partially scalar invariance for task value. Older adolescents scored lower on metacognitive self-regulation, mastery and performance goals, and self-efficacy. Older students scored lower on metacognitive self-regulation via indirect effects through Cholesky decomposed motivational factors.

**Discussion:**

Self-efficacy, mastery and performance goals, and task value are similarly understood across adolescents in different age groups. Decreased mastery and performance goals and task value can lead to reduced metacognitive self-regulation in adolescents. The implications of the findings underscore the key role of making students more engaged with lessons’ content in order to promote greater academic motivation and prevent decreases in metacognitive self-regulation.

## 1 Introduction

Metacognition, defined as students’ being aware and in control of their cognitive processes (Norman et al., 2019), is an critical antecedent of students’ academic success in school (de Boer et al., 2018; Katsantonis, 2020; Muncer et al., 2022). There is a controversy in the literature regarding the age differences in metacognition with some studies most disturbingly suggesting that adolescent students’ metacognition drops in secondary school (Wang and Eccles, 2012; Ahmed et al., 2013; Bardach et al., 2023), whereas other studies report increases in metacognition as students study in secondary school (Weil et al., 2013; dos Santos Kawata et al., 2021). One potential reason for decreased metacognitive skills might be related to students’ academic motivation. Students need to be motivated to effectively utilize metacognitive strategies in their learning (Zimmerman et al., 2017; de Boer et al., 2018). Holding greater levels of motivation has been linked with a manifold of positive outcomes, such as well-being (Howard et al., 2021), greater productivity (Cerasoli et al., 2014), and greater self-esteem (Tang et al., 2020). Nevertheless, research studies on adolescent students’ motivation and engagement in secondary schools have shown that students’ motivation and engagement are also declining (Wang and Eccles, 2013; Katsantonis and McLellan, 2023b; Katsantonis, 2024).

The above concerning evidence suggests that researchers need to examine in greater depth whether adolescent students’ academic motivation and metacognitive self-regulation are indeed declining in secondary schools. Moreover, based on past empirical evidence, it remains unclear which pathways lead to decreased metacognitive self-regulation, particularly considering that adolescents are expected to have developed improved metacognitive self-regulation from a developmental viewpoint (Veenman et al., 2006; Weil et al., 2013).

Therefore, the above raise the question whether students’ motivation and metacognitive self-regulation are indeed declining as students traverse through the different stages of lower secondary school. In the context of the present study, namely Greece, declines in students’ (aged ¬15 years) academic achievement have been noted over the years (Katsantonis et al., 2023; Katsantonis and McLellan, 2023b), which makes it more crucial than ever to examine whether decreased academic motivation as students study in higher grades is predictive of decreased metacognitive self-regulation, which is known to improve achievement. Overall, the present cross-sectional study’s purpose is twofold. First, to compare the motivation and metacognitive self-regulation levels of adolescents at different grades of lower secondary school education. Second, to examine potential mediating psychological mechanisms, whereby potential reductions in metacognitive self-regulation in language lessons occur through decreased self-efficacy, mastery and performance goals, and task value.

### 1.1 Age differences in metacognitive self-regulation

Although metacognition is made up by different facets such as metacognitive knowledge and experiences (Azevedo, 2020), the present study is focused on metacognitive self-regulation. Metacognitive self-regulation involves strategies for monitoring, controlling, and planning, which is a more higher-order metacognitive skill (de Boer et al., 2018; Katsantonis and McLellan, 2023a). However, research on age-related differences in metacognitive self-regulation has produced rather inconclusive and unintuitive findings, as will be shown.

Performance in experimental metacognitive tasks has been found to be higher in adolescence and dropping in adulthood (Weil et al., 2013). Additionally, research with self-report measures has also come to the same conclusion that adolescents have better metacognitive abilities (dos Santos Kawata et al., 2021). Evidence coming from longitudinal research has confirmed that the stage between 12 and 15 years is crucial for metacognitive development since the developmental trajectory of metacognitive skills is increasing between 13 and 14 years, but does not display a growth between 14 and 15 years (van der Stel and Veenman, 2014). Another comparative study showed contradictory findings. Specifically, this study reported that adolescents aged 14–15 years had better metacognitive self-regulation than adolescents aged 17–18 years (Bakracevic Vukman and Licardo, 2010). Additionally, studies have reported an overall decline in secondary school students’ metacognitive skills (Ziegler and Opdenakker, 2018; Bardach et al., 2023; Sáez-Delgado et al., 2023).

The findings of some studies that suggest decreased metacognitive capabilities in secondary schools is perplexing because metacognitive skills can be taught in schools (Perry et al., 2019). When metacognition is systematically trained, it can have a positive influence on students’ learning outcomes (de Boer et al., 2018). All the above contradicting evidence suggests that the study of age-related differences in metacognitive self-regulation, especially in connection with students’ secondary school grade level, is an area that requires further investigation. This raises the question: does metacognitive self-regulation indeed decrease as students traverse through higher grades of secondary school?

### 1.2 Age differences in academic motivation: self-efficacy, task value, and achievement goals

Academic or achievement motivation constitutes an umbrella term for various motivational factors that are typically linked with students’ achievement (Wigfield et al., 2021). There are various motivational factors recorded in the literature, such as self-efficacy, achievement goals, task value, flow, mindsets, etc. However, within the context of the cyclical self-regulated learning model (Zimmerman, 2008; Zimmerman et al., 2017), self-efficacy beliefs, task interest/value, and achievement goal orientations are considered important predictors of metacognitive self-regulation. Hence, the focus here is on these motivational factors.

Self-efficacy beliefs, defined as a self-belief of confidence in one’s capability to execute actions that will bring forth positive outcomes (Bandura, 1997), has been noted to face declines in adolescence. For instance, a longitudinal study with Italian adolescents revealed that self-efficacy beliefs declined between ages 12 and 18 years (Caprara et al., 2008). Other studies have also pointed toward age-related differences in self-efficacy with greater age being associated with reduced self-efficacy (Jacobs et al., 2002; Lee and Seo, 2021; Mozahem et al., 2021). Since the evidence is outdated, more recent empirical work should verify whether any age-related differences in adolescent self-efficacy are positive or negative.

Beyond self-efficacy, declines in task value have also been reported in the literature. Subjective task value refers to enjoying, liking or recognizing the instrumental value of a task or an activity (Eccles and Wigfield, 2020). For instance, a study with Korean adolescents reported average declines in both mathematics and language task value (Lee and Seo, 2021). However, more recent evidence has pointed toward a stable task value score throughout the adolescent years across multiple language and science domains (Guo et al., 2018; Part et al., 2023). Hence, more research is needed to verify how older students score in subjective task value.

Finally, the other important motivational factor is achievement goals. Achievement goals are broadly speaking distinguished between mastery (i.e., increasing effort and showing competence) and performance goals (i.e., outperforming others and selecting familiar tasks) (Lee and Bong, 2019). Adolescent students’ mastery goals’ levels have been found to drop in adolescence on average (Duchesne et al., 2014; Luo et al., 2023). Similarly, a drop in late adolescent (college students) performance goals has been reported in the past (Ciani et al., 2011; Liu et al., 2023). Given that some of the above evidence comes from late adolescent samples, it is reasonable to test whether any age differences in both mastery and performance goals exist with younger adolescents studying in secondary schools.

In brief, the declines in student motivation have been attributed in part, according to person-environment fit theory, to the structural changes in schools’ and classrooms’ attributes through the transition from primary to secondary school and throughout secondary school that result in person-environment mismatch (Eccles and Roeser, 2009; Wigfield et al., 2015). Hence, it might be likely that students’ academic motivation would decrease as academic demands increase as students attend more advanced grades in secondary school.

### 1.3 Conceptual framework linking academic motivation with metacognitive self-regulation

The structural links between academic motivation and metacognitive self-regulation are complicated. Theoretical support for the connection between academic motivation and metacognitive self-regulation comes from the self-regulated learning (SRL) theory. SRL theoretical models suggest that cognitive, motivational, metacognitive, affective, and behavioral factors all come together to shape students’ learning (Efklides, 2019; Zeidner and Stoeger, 2019). The cyclical model of SRL indicates that SRL is taking place in three ordered phases, called forethought, performance, and self-reflection, that reflect the causal links between SRL processes and academic motivation (Callan and Cleary, 2019). In the cyclical SRL model it is generally understood that academic motivation (i.e., self-efficacy, goal orientations, and task value) typically serves as an antecedent of metacognitive self-regulation (Zimmerman and Moylan, 2009; de Boer et al., 2018; Katsantonis and McLellan, 2023a). However, the links between the different motivational factors are unclear and the existing studies usually disagree regarding the directional nature of these associations (e.g., Chatzistamatiou et al., 2015; Cleary and Kitsantas, 2017; Katsantonis et al., 2023). Additionally, it is yet not clear in the literature which academic motivation factor contributes most to metacognitive self-regulation. Hence, in this study a Cholesky decomposition is (de Jong, 1999) deployed to study the independent contribution of each of the above motivational factors above and beyond each other to metacognitive self-regulation.

**Figure 1:**
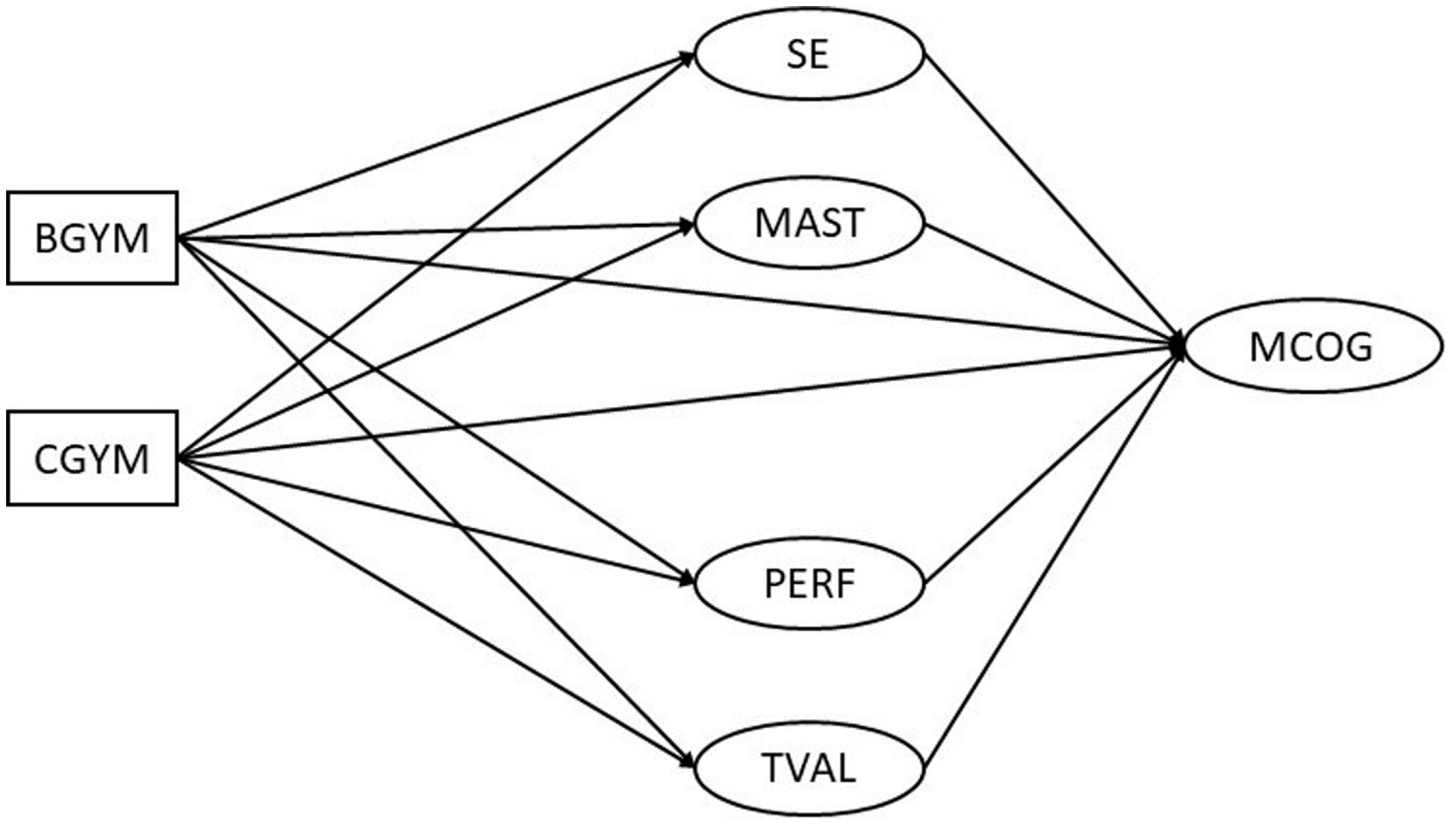
Conceptual model showing the mediating role of academic motivation factors in the relation between grade membership in lower secondary school and metacognitive self-regulation. SE, self-efficacy; MAST, mastery goals; PERF, performance goals; TVAL, task value; MCOG, metacognitive self-regulation; BGYM, B Gymnasium (second grade of Gymnasium); CGYM, C Gymnasium (third grade of Gymnasium).

Given that the theoretical and empirical evidence regarding the declines in metacognitive skills of the adolescents is rather mixed, it might be possible that any decreases in metacognitive self-regulation might be related to reduced academic motivation. Hence, the current study explores this possibility through a mediation model, whereby students’ age (by proxy of grade membership) is predicting the motivational factors, which, in turn, predict metacognitive self-regulation. This conceptual model is presented in Figure 1.

### 1.4 An overview of the Greek educational system and language learning in lower secondary schools

The Greek educational system is centralized, which means that the Ministry of Education is the highest authority for administrative and pedagogical matters (Kougias and Efstathopoulos, 2020; Katsantonis et al., 2023). In this system, schools and teachers have limited autonomy to implement their own policies and pedagogical practices. This is because the system is centralized and requires uniformity from the allocation of funds to school curriculums, textbooks, and policies concerning teachers and students (Persianis, 2003). Education in Greece is compulsory for all children from age 4 to age 15. There are three broad key stages of education, namely kindergarten, primary school, and secondary school (Giamouridis and Bagley, 2006). Secondary school, which is the focus of this study, is further divided into two broad cycles of education called Gymnasium (lower secondary school-ages 12–15) and Lyceum (upper secondary school-ages 15–18) (Giamouridis and Bagley, 2006). Only Gymnasium is part of the compulsory education. Gymnasiums are made up by three grades, namely A, B, and C Gymnasium.

Modern Greek language lessons are compulsory in every grade and take place at least twice per week (Ministry of Education, 2022). The purposes of the lessons are to teach students skills such as reading and comprehending written and oral speech, identification of text genres, the acquisition of the structure of Greek language, and the function of grammatical phenomena in texts, and the production of written texts, among others (Ministry of Education, 2022). Progressing through the grades of the Gymnasium, students increase their acquisition of skills and knowledge that they have already acquired in earlier grades (Greek Government, 2022). Both the language curriculum and the assessment methods include the aim of improving students’ metacognitive skills to ensure the comprehension and interpretation of concepts, phenomena, and processes through the control and regulation of their learning (Greek Government, 2022). Nevertheless, Greek adolescent students are known to perform badly in international comparative studies of students’ language skills in the last decades (Katsantonis and McLellan, 2023a). This makes it more crucial than ever to examine whether students’ metacognitive self-regulation drops as they study in higher grades of secondary school since metacognitive self-regulation is such an important predictor of achievement (de Boer et al., 2018).

### 1.5 The present study

The potential decreases in metacognitive self-regulation as students study in higher grades of secondary school is concerning and requires further study. Therefore, the current study aims to examine whether adolescents’ motivation and metacognitive self-regulation drop in secondary schools in Greece using a comparative cross-sectional approach. To address this aim, the present study goes beyond simple comparisons between observed mean scores across groups of adolescents and examines if different age groups construe the psychological meaning of the different academic motivation factors and metacognitive self-regulation similarly. Second, the study puts forward a plausible explanatory mechanism of the potential drop in metacognitive self-regulation via academic motivation factors. Instead of testing a conventional mediation model given the assumed high intercorrelations between the motivational beliefs, the present study employs the advanced Cholesky decomposition (de Jong, 1999) to examine this mediational pathway through the independent contribution of the different motivational factors. In brief, the following research questions are addressed in this study.

*RQ1*: Are the different motivational factors and metacognitive self-regulation measurement invariant across adolescents belonging to different grades?

*RQ2*: How do different groups of adolescent lower secondary school students score in metacognitive self-regulation and the different motivational factors?

*RQ3*: Do the different motivational factors serve as independent mediators between students’ age and metacognitive self-regulation above and beyond the other motivational factors?

## 2 Materials and methods

### 2.1 Research methodology

The current study follows the principles of survey research (Cohen et al., 2018) to understand the factors that are associated with the language achievement of students in lower secondary schools in Greece. The instrument of data collection was a structured questionnaire that asked students to report information on their demographics and respond to several items about their motivation, metacognitive self-regulation, achievement, and outcome expectancies. This study is part of larger project (Katsantonis and McLellan, 2023a), whose data collection protocols have received ethics approval from the Faculty of Education, University of Cambridge, United Kingdom. The study was conducted after gaining approval from the Greek Ministry of Education. Students were appraised of the content of the survey questionnaire and parents/legal guardians signed informed consent forms. The data were collected between December 2022 and late April 2023.

### 2.2 Participants

The participants of this study comprise 1,027 adolescent students (ages 12–16, *M*_age_ = 13.95, *SD* = 0.78). Participants were studying in the first (A Gymnasium), second (B Gymnasium), or third (C Gymnasium) grade of Greek lower secondary schools and came from a range of socio-economic backgrounds. The data were collected from 19 schools. The sample was made up by 46.71% male students and 53.29% female students. The ages of the students in years are distributed as follows 12-years (5.77%), 13-years (15.17%), 14-years (57.53%), 15-years (20.74%), and 16-years (0.78%). From these students, 106 (10.30%) were studying in A Gymnasium, 376 (36.54%) were studying in B Gymnasium, and 545 (52.96%) were studying in C Gymnasium.

### 2.3 Measures

All measures here come from the Motivated Strategies for Learning Questionnaire (MSLQ) (Pintrich et al., 1991; Pintrich, 2003), which has been successfully used in the past with even younger samples from primary schools in Greece (Andreou and Metallidou, 2004; Metallidou and Vlachou, 2007). The MSLQ is a well-validated questionnaire that has been used around the world (Duncan and McKeachie, 2005; Credé and Phillips, 2011).

#### 2.3.1 Language lesson metacognitive self-regulation

The nine items from the metacognitive self-regulation scale of the MSLQ measure planning, monitoring, and control of cognition (Pintrich et al., 1991). The question prompt and the items were slightly adapted to refer to the language lessons in Greek schools. Given the known latent factor structure of this scale, the three negatively worded items were dropped from the analyses due to a method factor (Tock and Moxley, 2017). A sample item from this scale is “I work on practice exercises and answer end of chapter questions even when I do not have to.” A rating scale ranging between 1 = “not at all true of me” and 7 “very true of me” was used. McDonald’s omega coefficient for this scale was good, *ω* = 0.85. Item-total correlations ranged from 0.49 to 0.61, indicating very good psychometric quality (Cristobal et al., 2007).

#### 2.3.2 Language lesson self-efficacy

The nine items of the academic self-efficacy for learning and performing scale of the MSLQ were administered (Pintrich, 2003). The question prompt was slightly modified to refer to language learning and performing in the Greek language lessons. A sample item is “I’m certain I can understand the ideas taught in this course.” A rating scale ranging between 1 = “not at all true of me” and 7 “very true of me” was used. McDonalds’ omega coefficient of reliability for this scale was also very good, *ω* = 0.92. Item-total correlations ranged from 0.61 to 0.74.

#### 2.3.3 Language lesson mastery goal

Four items comprise the mastery goal scale of the MSLQ (Pintrich et al., 1991). A sample item was “In a class like this, I prefer course material that arouses my curiosity, even if it is difficult to learn.” A rating scale ranging between 1 = “not at all true of me” and 7 “very true of me” was used. McDonald’s omega indicated very good reliability, *ω* = 0.75. Item-total correlations ranged from 0.33 to 0.51.

#### 2.3.4 Language lesson performance goal

Performance goals were measured using the four items of the extrinsic goals scale of the MSLQ (Pintrich et al., 1991). A sample item for this scale is “If I can, I want to get better grades in this class than most of the other students.” A rating scale ranging between 1 = “not at all true of me” and 7 “very true of me” was used. McDonald’s coefficient omega indicated very good reliability, *ω* = 0.75. Item-total correlations ranged from 0.47 to 0.56.

#### 2.3.5 Language lesson task value

The final scale that was administered to students was the six items-long task value scale of the MSLQ (Pintrich et al., 1991). This scale was slightly modified to refer to the Greek language lesson. The scale captures students’ opinions about their intrinsic interest in Greek language lessons and the instrumental value of the lessons. A sample item is “I am very interested in the content area of this lesson.” A rating scale ranging between 1 = “not at all true of me” and 7 “very true of me” was used. McDonald’s omega coefficient was excellent for this scale, *ω* = 0.93. Item-total correlations ranged from 0.67 to 0.78.

#### 2.3.6 Students’ grade membership

Students reported on their current grade membership. This was an ordinal-categorical variable ranging from 0 to 2, whereby 0 was A Gymnasium, 1 was B Gymnasium, and 2 was C Gymnasium. Higher grade membership indicated that the students were older and studied in a more advanced grade in lower secondary school. Grade membership is utilized in this study as a proxy for age since it nicely clusters students together and clearly reflects their educational stage and learning age. The use of grade as a proxy for age is common in educational psychology studies (Li and Lerner, 2011; Ansari et al., 2020).

#### 2.3.7 Students’ sex

A binary variable reflecting whether students were female or male.

### 2.4 Data analyses

In the first instance, McDonald’s reliability coefficient omega was calculated (McDonald, 1999) and item-total correlations were computed. Omega values above 0.70 and item-total correlations above 0.30 are considered to reflect very good reliability (cf., Cristobal et al., 2007; McNeish, 2018). Latent bivariate correlations and descriptive statistics were calculated to inspect the patterns of the data. The suitability of the data for multilevel modeling was examined using the intra-class correlation coefficient (ICC), whereby ICC values less than 5% suggest that multilevel modeling is not appropriate (Dyer et al., 2005). Afterwards, multigroup measurement invariance analyses were performed with students’ grade membership as the grouping variable (*n* = 106 students in A Gymnasium; *n* = 376 students in B Gymnasium; *n* = 545 students in C Gymnasium). Using grade membership as the grouping variable for testing age differences, aside from the fact that it creates clearly distinct groups, it is very common in educational and developmental psychology studies since it clusters together students that have similar educational and learning levels (Bong, 2009; Lee and Seo, 2021). The measurement invariance analyses permit researchers to ascertain whether the psychological measures are similarly construed across grade groups and whether any either observed or latent mean differences are entirely attributable to the latent factor (Putnick and Bornstein, 2016; Kline, 2023). The levels of measurement invariance testing are described elsewhere (Vandenberg and Lance, 2000). However, it should be noted that achieving metric invariance permits accurate and unbiased comparisons of *latent* correlations and regression coefficients but not the observed correlations and regression coefficients (Gregorich, 2006). Scalar invariance permits direct comparisons of the latent and observed means (Gregorich, 2006; Sass, 2011). Failure to achieve full invariance at any level, does not necessarily mean a termination of invariance testing. The analysts can pursue partial invariance, whereby some item’s factor loading or intercept/thresholds can be freely varying across groups following the guidance of the modification indices (Byrne et al., 1989).

Having tested the invariance of the five scales across the age groups (by proxy of grade membership), a Cholesky decomposition model was implemented (de Jong, 1999; Bentler and Satorra, 2000), which is akin to a hierarchical regression analysis in the structural equation framework. The Cholesky decomposition allows the estimation of the independent contribution of each motivational factor to metacognitive self-regulation and controls for potential multicollinearity between the variables (de Jong, 1999). To achieve these aims, phantom factors are introduced that capture the correlations between the motivational latent factors (de Jong, 1999). Four uncorrelated latent factors, called Cholesky factors, were created with their variances fixed to unity for identification (de Jong, 1999). The factor loadings of the Cholesky factors were freely estimated (de Jong, 1999). For this study, the entry into the model is: (a) Mastery goals; (b) Performance goals; (c) Task value; (d) Self-efficacy. So, the fourth Cholesky factor (Ch4) predicts all academic motivation factors. Next, mastery goals are removed from the third Cholesky factor (Ch3) reflecting the influence of performance goals. Afterwards, performance goals are removed from the second Cholesky factor (Ch2), reflecting, thus, the influence of task value. Finally, only self-efficacy loads on the first Cholesky factor (Ch1), reflecting, thus, the influence of self-efficacy net from the other motivational factors. The square of the beta coefficients indicates the proportion of explained variance (Δ*R*^2^) in metacognitive self-regulation by each motivational factor (de Jong, 1999). The advantage of the Cholesky method in structural equation modeling is that it controls for measurement error, which standard ordinary least squares regression cannot do (Kline, 2023).

To test the mediating effect of the motivational factors between grade grouping and metacognitive self-regulation, students’ grade membership was recoded as two binary dummy variables with the A Gymnasium as the reference group. Hence, students studying in B Gymnasium and C Gymnasium were compared to the students studying in A Gymnasium. This is a preferable analytic choice since the sample size in A Gymnasium was rather smaller and would have been underpowered for such a large structural model.

Turning now to matters of model-data fit, the conventional cut-offs in the goodness-of-fit indices were considered here. Specifically, CFI and TLI values close to/above 0.95, accompanied by an RMSEA value below 0.06 and a SRMR value below 0.08 are considered indicators of good fit (Hu and Bentler, 1999). The chi-square test is usually very sensitive to minor misspecifications and was, thus, not of primary interest here given the large sample size (Bearden et al., 1982). To evaluate measurement invariance, the Satorra-Bentler chi-square differences test (Satorra and Bentler, 2001) was utilized along with CFI and RMSEA cut-offs of 0.01 and 0.015, respectively (Chen, 2007). The latent factor means were compared using the standardized mean differences (SMD) effect size, whereby values of SMD = 0.2 are small; values of SMD = 0.5 are medium; and values of SMD = 0.8 are large (Cohen, 1988). All models were estimated using robust standard errors via the robust maximum likelihood estimator (MLR). Missing data were handled using the full-information maximum likelihood method (Enders, 2022). All structural equation modeling was performed in Mplus 8.7 (Muthén and Muthén, 2017). Indirect effects were estimated using the MODEL INDIRECT command in Mplus. McDonald’s omega coefficient of reliability was estimated using the psych package (Revelle, 2022) in R (R Core Team, 2023).

## 3 Results

### 3.1 Descriptive statistics and bivariate latent correlations

Descriptive statistics and latent bivariate correlations between the key outcomes and covariates were calculated first and are presented in Table 1. The intra-class correlation coefficients for the key variables were extracted from an intercept-only multilevel model and were found to be less than 5%. This suggests that multilevel modeling is not required since the school-level explains very little variance in metacognitive self-regulation and academic motivation factors (Hox et al., 2017). Missing data analysis revealed only 9.72% of missing values. Little’s MCAR test was statistically significant (*p* < 0.001) for the key outcomes suggesting that the data were not missing completely at random (Little, 1988). Accounting for students’ sex, the MCAR test became statistically insignificant (*p* > 0.05), indicating that the data were conditionally missing. From the latent correlation matrix (Table 1), it becomes clear that some motivational factors are quite strongly correlated. Therefore, the Cholesky decomposition appears to be a reasonable modeling choice.

**Table 1:** Descriptive statistics and latent bivariate correlations.

Variable	1	2	3	4	5	6	7
1. Sex	1						
2. Grade	0.121^***^	1					
3. MCOG	0.219^***^	−0.201^***^	1				
4. SE	0.165^***^	−0.123^***^	0.666^***^	1			
5. MAST	0.243^***^	−0.066	0.713^***^	0.698^***^	1		
6. PERF	0.045	−0.162^***^	0.448^***^	0.379^***^	0.340^***^	1	
7. TVAL	0.250^***^	−0.155^***^	0.752^***^	0.633^***^	0.797^***^	0.415^***^	1
Descriptive statistics							
*M* (*SD*)	1.532 (0.50)	1.427 (0.67)	25.65 (7.97)	44.18 (10.41)	19.03 (4.98)	20.46 (5.32)	27.56 (8.61)
Min–Max	1–2	0–2	6–42	9–63	4–28	4–28	6–42
ICC			0.036	0.016	0.018	0.042	0.045

***p < 0.001, SEX, female vs. male; Grade, students’ grade membership in secondary school; MCOG, metacognitive self-regulation; SE, self-efficacy; MAST, mastery goals; PERF, performance goals; TVAL, task value; Descriptive statistics refer to computed summed composite scores; Min, minimum observed score; Max, maximum observed score; M, mean: SD, standard deviation; One residual correlation was introduced between two items of the metacognitive self-regulation scale; ICC, intra-class correlation coefficient for school-level; Two residual correlations were introduced in the task value scale.

### 3.2 Multigroup measurement invariance analyses: testing age-related mean differences in motivation and metacognitive self-regulation

In multigroup measurement invariance analyses, students’ grade membership was used as the grouping variable since this created clear groupings of students. Three levels of invariance are tested, namely configural, metric, and scalar, and the models were compared to determine what level of invariance was tenable. The invariance analyses’ results are presented in Table 2. As shown in Table 2, metacognitive self-regulation, self-efficacy, and mastery and performance goals were scalar invariant across age groups both according to the Satorra-Bentler chi-square differences test and to the approximate fit indices. However, the task value scores were not fully scalar invariant, but partially scalar invariance was achieved by releasing the equality constraints on the two final items of the scale (i.e., “I like the subject matter of this lesson” and “understanding the subject matter of this lesson is important for me”) for the C Gymnasium group.

**Table 2:** Multigroup measurement invariance analyses’ results-comparisons between invariance levels.

Invariance level	SB Δ*χ*^2^ (df)	CFI	|ΔCFI|	RMSEA	|ΔRMSEA|
	Language lesson metacognitive self-regulation				
Configural		0.993		0.029	
Metric	5.398 (10)^ns^	0.997	0.004	0.016	0.013
Scalar	14.024 (10)^ns^	0.993	0.004	0.021	0.005
	Language lesson self-efficacy				
Configural		0.950		0.068	
Metric	13.816 (16)^ns^	0.947	0.003	0.064	0.004
Scalar	25.242 (16)^ns^	0.941	0.006	0.062	0.002
	Language lesson mastery goal				
Configural		0.987		0.056	
Metric	7.345 (6)^ns^	0.984	0.003	0.043	0.013
Scalar	8.442 (6)^ns^	0.979	0.005	0.041	0.002
	Language lesson performance goal				
Configural		0.987		0.056	
Metric	7.345 (6)^ns^	0.984	0.003	0.043	0.013
Scalar	8.442 (6)^ns^	0.979	0.005	0.041	0.002
	Language lesson task value				
Configural		0.985		0.062	
Metric	15.270 (10)^ns^	0.981	0.004	0.057	0.005
Scalar	42.406 (10)^***^	0.966	0.015	0.066	0.009
Partially scalar	7.595 (8)^ns^	0.979	0.002	0.053	0.004

***p < 0.001; ns, not statistically significant; SB, Satorra–Bentler; partially scalar model had relaxed the intercepts of two items in the C Gymnasium group.

**Figure 2:**
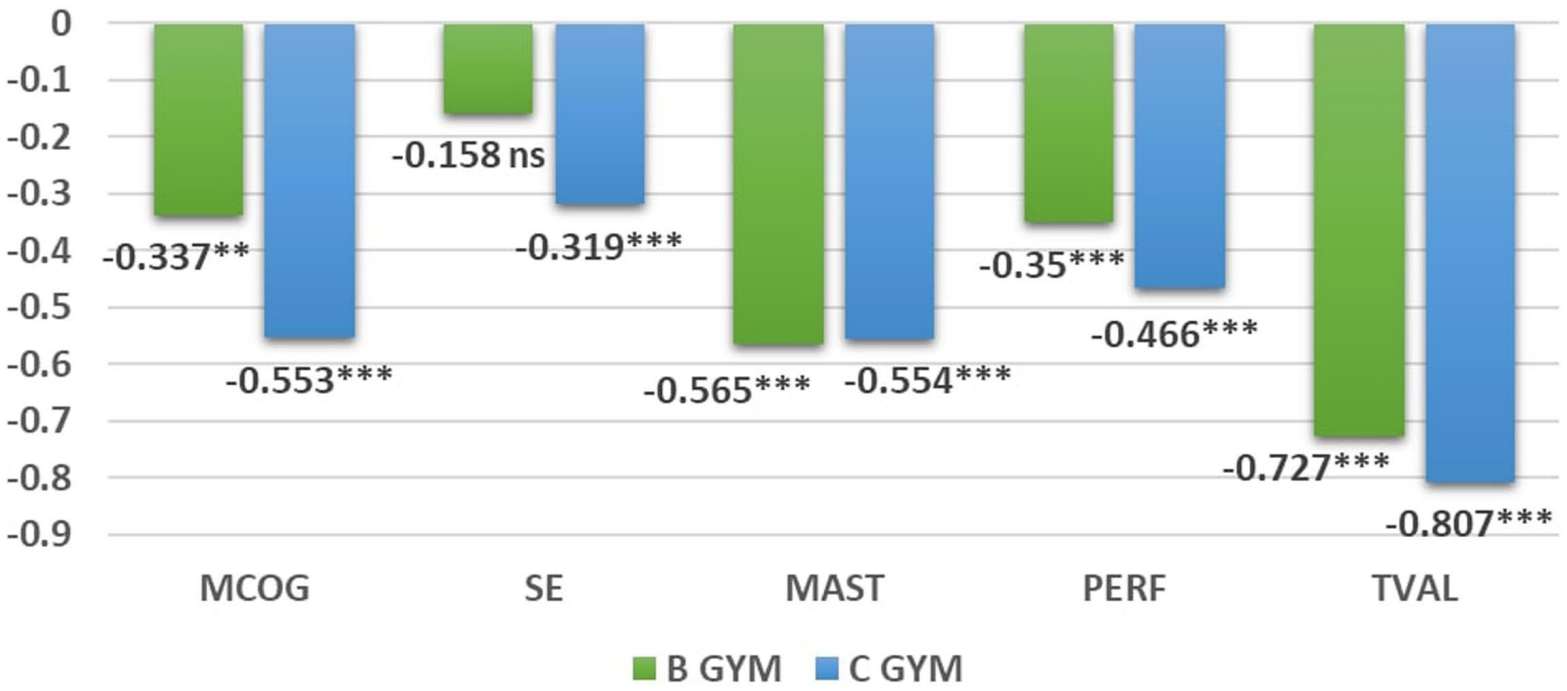
Standardized latent factor mean differences in metacognitive self-regulation and motivational variables. MCOG, metacognitive self-regulation; SE, delf-efficacy; MAST, mastery goal; PERF, performance goal; TVAL, task value; B Gym, B Gymnasium; C GYM, C Gymnasium; ***p < 0.001, **p < 0.01; *ns*, not statistically significant; all latent means are in comparison to A Gymnasium students.

Following the invariance testing analyses, the standardized latent factor means were compared to the reference group, which is the A Gymnasium group. The latent SMDs are presented in Figure 2. Small between-group standardized mean differences were found between A Gymnasium and B Gymnasium in metacognitive self-regulation and performance goal, suggesting a small decrease for B Gymnasium in these domains. Moderate differences were found between A Gymnasium and B Gymnasium in task value and mastery goals. Moderate standardized differences were found between A Gymnasium and C Gymnasium in mastery goal, metacognitive self-regulation, and performance goals. Finally, large differences occurred between A Gymnasium and C Gymnasium in task value. In brief, decreases in all motivational factors and metacognitive self-regulation were found as students became older and studied in more advanced grades in lower secondary schools.

### 3.3 Motivational mechanisms underpinning decrements in metacognitive self-regulation

**Figure 3:**
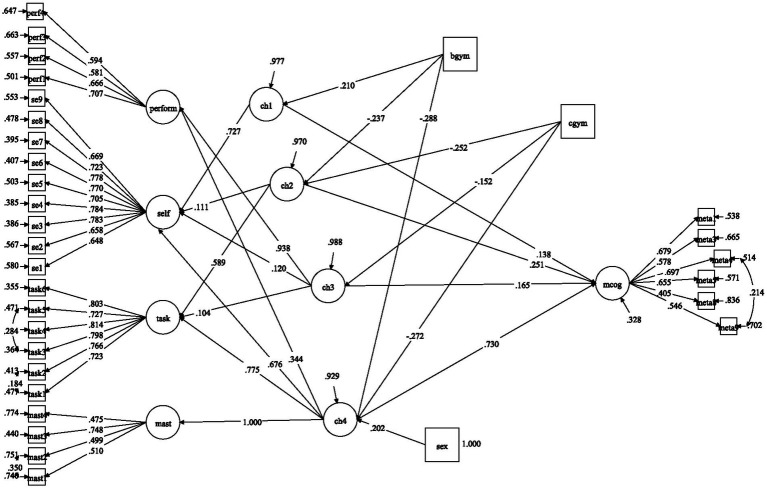
Full structural model of Cholesky decomposition factors predicted by grade membership and sex and predicting metacognitive self-regulation. bgym, B Gymnasium; cgym, C Gymnasium; sex, female vs. male; mcog, metacognitive self-regulation; mast, mastery goals; task, task value; self, self-efficacy; perform, performance goals; ch1–ch4, Cholesky factors; CH4, Cholesky factor capturing the net effect of mastery goals; CH3, Cholesky factor capturing the net effect of performance goals; CH2, Cholesky factor capturing the net effect of task value; CH1, Cholesky factor capturing the net effect of self-efficacy. Only statistically significant standardized effects depicted (at least *p* < 0.05).

The results of multigroup measurement invariance analyses indicated an overall decline in adolescent students’ motivation and metacognitive self-regulation. Yet, it is not clear what is the mechanism underpinning these declines in metacognitive self-regulation. Hence, a multiple mediation model via the Cholesky factors was tested. However, before the full mediation model was tested, a direct effects-only model from students’ grade membership to metacognitive self-regulation was tested first. This model revealed statistically significant direct effects from the dummy variables to metacognitive self-regulation, *β*_BGYM_ = −0.161, *p* < 0.01, and *β*_CGYM_ = −0.268, *p* < 0.001. Afterwards, the full mediation was tested (see Figure 3). This final model had a reasonably good fit to the data with CFI = 0.932, TLI = 0.923, RMSEA = 0.039 90%CI [0.037, 0.042], SRMR = 0.048.

As shown in Figure 3, several important findings occurred. First, the direct effects from B Gymnasium and C Gymnasium to metacognitive self-regulation, *β*_BGYM_ = 0.082, *p* > 0.05, and *β*_CGYM_ = −0.022, *p* > 0.05, respectively, did not reach statistical significance. The first Cholesky factor, which captured the variance in self-efficacy, was positively predicted by B Gymnasium but not by C Gymnasium. The addition of task value in the second Cholesky factor positively predicted metacognitive self-regulation (*β* = 0.251, *p* < 0.001) but was negatively predicted by both B and C Gymnasium. The third Cholesky factor included additionally performance goals and positively predicted metacognitive self-regulation (*β* = 0.165, *p* < 0.001), but was negatively predicted only by C Gymnasium. Finally, the fourth Cholesky factor included mastery goals and positively predicted metacognitive self-regulation (*β* = 0.730, *p* < 0.001), but was negatively predicted by B and C Gymnasium. From the Cholesky effects it became apparent that mastery goals were the strongest predictor of metacognitive self-regulation explaining 53%. The addition of performance goals explained an additional 3%, whereas the addition of task value explained an additional 6.2%. Finally, self-efficacy explained an additional 2%. Overall, the full model explained an impressive 67.2% of the variance in metacognitive self-regulation.

The specific indirect effects arising from the full structural mediation model were computed and are presented comprehensively in Table 3. As discussed above, higher grades of students did not have a direct effect after introducing the motivational Cholesky factors. In Table 3, the reduction in metacognitive self-regulation for older students is observed via a reduction in task value, performance goals, and, especially, mastery goals. Therefore, a reasonable conclusion is that the reduction in metacognitive self-regulation is possibly the by-product of a reduced motivation in the language lesson and its content material.

**Table 3:** Standardized specific indirect regression effects derived from the full structural model.

Indirect effect	*β* (*S.E.*)	Two-tailed *p*-value
BGYM → CH1 → MCOG	0.029 (0.011)	0.007
CGYM → CH1 → MCOG	0.011 (0.008)	0.174
BGYM → CH2 → MCOG	−0.060 (0.022)	0.006
CGYM → CH2 → MCOG	−0.063 (0.022)	0.003
BGYM → CH3 → MCOG	−0.011 (0.010)	0.264
CGYM → CH3 → MCOG	−0.025 (0.012)	0.033
BGYM → CH4 → MCOG	−0.210 (0.049)	0.000
CGYM → CH4 → MCOG	−0.198 (0.049)	0.000

β, linear regression coefficient; S.E., standard error; BGYM, B Gymnasium; CGYM, C Gymnasium; CH1-CH4, Cholesky factors; MCOG, metacognitive self-regulation; CH4, Cholesky factor capturing the net effect of mastery goals; CH3, Cholesky factor capturing the net effect of performance goals; CH2, Cholesky factor capturing the net effect of task value; CH1, Cholesky factor capturing the net effect of self-efficacy.

## 4 Discussion

Given the importance of metacognitive self-regulation skills for students’ academic achievement (de Boer et al., 2018; Katsantonis and McLellan, 2023a,b), the present study examined age differences in metacognitive self-regulation skills and the potentially mediating role of students’ motivational beliefs. The purpose of the study was to gain greater insights into the mechanisms that underpin students’ declining metacognitive self-regulation skills as students studied in more advanced grades.

The first objective of this study was to examine the possible reductions in motivational factors (i.e., self-efficacy, achievement goals, and task value) and metacognitive self-regulation. The results of multigroup measurement invariance analyses between students’ grades revealed that adolescent students have similar understanding of the psychological meaning of the motivational factors and metacognitive self-regulation across the different grade groups. Comparisons of the latent factor means revealed an average drop in academic self-efficacy, task value, and achievement goals with an increase in age, as students studied in higher grades. This finding is to some extent compatible with past evidence suggesting a decline in academic self-efficacy (Caprara et al., 2008; Lee and Seo, 2021; Mozahem et al., 2021), achievement goals (Ciani et al., 2011; Duchesne et al., 2014; Luo et al., 2023), and task value (Watt, 2004; Lee and Seo, 2021).

However, some methodological differences should be noted here since they outline the contribution of the current study. First, the present study examined age group differences in early and middle adolescence (Salmela-Aro, 2011). In contrast, some past studies have focused on late adolescents/emerging adults (Ciani et al., 2011; Guo et al., 2018; Liu et al., 2023). Additionally, the present study ensured that the psychometric measures were equivalently construed and measured across the different age groups (by proxy of grade membership), which is something that has not be examined in most of the past evidence (Caprara et al., 2008; Ciani et al., 2011; Liu et al., 2023). Hence, the present findings provide more nuanced evidence of mean differences. The robust negative mean differences across grades in secondary school suggest that students in higher grades are feeling less confident in their capabilities (self-efficacy), have less intrinsic and instrumental value for the language lesson (task value), and are less interested in displaying mastery and performance goals.

Beyond the findings of academic motivation declines with increased grade membership, the present study contributes to ongoing debates about the age differences in metacognitive self-regulation skills. The current study’s findings indicate a decrease in adolescent students’ metacognitive self-regulation skills in the language lesson, as students study in higher grades in secondary school. Therefore, the findings corroborate with past evidence indicating a decline in metacognitive skills in adolescence and, particularly, in secondary school (Ahmed et al., 2013; Bardach et al., 2023). However, the current findings contradict the other research strand that suggested that metacognitive skills become more refined in adolescence (Veenman et al., 2006; Weil et al., 2013; dos Santos Kawata et al., 2021). This is a particularly concerning finding since it shows that older students studying in higher grades in secondary school are reporting to be less effective in metacognitive self-regulation strategies that could assist them in becoming better achievers in school. The fact that metacognitive self-regulation strategies (Cer, 2019; Perry et al., 2019) can be effectively taught but students report decreased metacognitive self-regulation strategies in higher grades of lower secondary schools suggests that there might be an issue with the teaching quality or the curriculum structure is not appropriate for fostering such strategies.

Yet, the motivational mechanism that might explain this decreased metacognitive self-regulation in secondary schools is a relatively under-researched topic. Hence, drawing upon the cyclical model of self-regulated learning (Zimmerman, 2008; Zimmerman and Moylan, 2009), the hypothesis was that decreased motivation would propagate the negative effect of grade membership to metacognitive self-regulation. To examine this mechanism, a structural equation model with Cholesky decomposed motivational factors was estimated. The findings revealed new insights into the decreased metacognitive self-regulation. Specifically, grade differences in metacognitive self-regulation were negated once the Cholesky decomposed motivational factors were introduced into the model. The results of the full model indicated that only specific motivational factors can be linked with decreases in metacognitive self-regulation. For instance, being older and studying in the B Gymnasium was associated with greater self-efficacy and, subsequently, greater metacognitive self-regulation net of other motivational factors. However, being older and studying in a higher grade was associated with less task value, and less mastery and performance goals, which propagated a negative indirect effect on metacognitive self-regulation. This suggests that the declines in metacognitive self-regulation latent means can be partially explained by the declines in students’ motivational beliefs as students become older and study in higher grades in lower secondary school. Reductions in students’ mastery goals appeared to be the most significant explanatory factor since mastery goals explained 53% of the variance. To some extent, the predictive relation between the different motivational factors and metacognitive skills has already been noted (Coutinho and Neuman, 2008; Chatzistamatiou et al., 2015; Katsantonis, 2020). Nevertheless, the fact that the age differences in metacognitive self-regulation can be explained to a great extent indirectly through the age differences in academic motivation is a new contribution to the field.

### 4.1 Strengths, limitations, and future directions

As with all studies, the present investigation was also characterized by some strengths and limitations. First, the sample size was sufficiently large and covered a range of schools that, despite not being representative, makes it more inclusive of different student characteristics. Second, the measures utilized in this study are well-validated and have been found to work well in the past. Third, the study’s design was cross-sectional, which means that differences between the different groups of students could also reflect differences in their other sample characteristics. However, in supplemental regression analyses, which are available upon request, controlling for gender and socio-economic status, grade differences remained statistically significant. Given that the cross-sectional nature of the study’s design prohibits causal conclusions, more longitudinal research studies in this field are needed. Specifically, longitudinal growth curve models in combination with cross-lagged panel models will be appropriate methods to confirm these findings. Finally, new online methods could be utilized to gain deeper insights into metacognitive self-regulation declines.

### 4.2 Implications

Both metacognitive self-regulation and students’ academic motivation are important factors closely tied to students’ learning and achievement (Hattie, 2010). It is important to enhance secondary school students’ motivation and metacognitive self-regulation skills in lower secondary schools, especially in higher grades when students are more vulnerable to reduced motivation and metacognitive self-regulation. This could be especially important for students studying in higher grades, who score lower on these measures. Improving students’ motivation could be achieved through curriculum change or via teachers’ agency, whereby teachers will adopt more student-centric approaches to adapt the learning materials to students’ interests. Metacognitive self-regulation might be improved through the implementation of explicit teaching or through specific interventions (Perry et al., 2019). Systematic teaching of planning, monitoring, and cognitive control strategies is particularly important because metacognitive skills should be more refined in this period, rather than being reduced. This suggests that the teaching quality needs to be higher or the students should be more attentive and actually implement such strategies in language lessons. Since mastery goals were most strongly associated with metacognitive self-regulation, it is recommended that schools place emphasis on students exhibiting their competence in language lessons by acquiring new skills. The fact that self-efficacy was not associated with decreased metacognitive self-regulation, controlling for the other motivational factors, suggests that learning experiences that boost students’ self-efficacy could have a beneficial effect on planning, monitoring, and cognitive control strategies in language lessons.

## 5 Conclusion

In conclusion, this study examined age differences in adolescent academic motivation and metacognitive self-regulation. Substantial differences were detected between three groups of students studying in different grades in lower secondary schools in Greece. Older students in higher grades had worse self-efficacy, task value, mastery and performance goals, as well as lower metacognitive self-regulation. Decreased task value, mastery and performance goals were propagating the negative effect of age on metacognitive self-regulation, suggesting that motivation is a possible leading factor in declining metacognitive self-regulation in adolescent students.

## Data availability statement

The raw data supporting the conclusions of this article will be made available by the authors, without undue reservation.

## Ethics statement

The studies involving humans were approved by Ethics Committee at the Faculty of Education, University of Cambridge, UK (29/7/2022). The studies were conducted in accordance with the local legislation and institutional requirements. Written informed consent for participation in this study was provided by the participants’ legal guardians/next of kin.

## Author contributions

IK: Conceptualization, Data curation, Formal analysis, Funding acquisition, Investigation, Methodology, Project administration, Resources, Software, Supervision, Validation, Visualization, Writing – original draft, Writing – review & editing.
