# Supplementary material for: Exploring age-related differences in metacognitive self-regulation: the influence of motivational factors in secondary school students
Source: Front Psychol. 2024 Jun 25;15:1383118. doi: 10.3389/fpsyg.2024.1383118 (PMC11231430; doi:10.3389/fpsyg.2024.1383118)
Supplement: Supplementary file 1 [file Table_1.DOCX]

Supplementary Material

# Supplementary Data

**Motivated Strategies for Learning Questionnaire** (Pintrich et al., 1991)

The following questionnaire contains some questions regarding your self-confidence in the subject of **Modern Greek Language**, your motivations for learning, and the strategies you use to better understand this subject. The questionnaire is anonymous, and there are no right or wrong answers. Please respond as honestly as you can - your parents, classmates, and teachers will not see your answers.

Please mark with **x** in the box according to whether you agree or disagree with the following statements. 1 corresponds to "Not applicable at all for me," and 7 corresponds to "Very applicable to me. You can choose any number between 1 and 7.

**S1 Table. Academic self-efficacy in school language class**

| 1. Compared with other students in this **class** I expect to do well |
| --- |
| 1. I'm certain I can understand the ideas taught in this course |
| 1. I expect to do very well in this class |
| 1. Compared with others in this class, I think I'm a good student |
| 1. I am sure I can do an excellent job on the problems and tasks assigned for this class |
| 1. I think I will receive a good grade in this class |
| 1. My study skills are excellent compared with others in this class |
| 1. Compared with other students in this class I think I know a great deal about the subject |
| 1. I know that I will be able to learn the material for this class |

Class was substituted with modern Greek language class throughout the questionnaire. The word “course” was changed to modern Greek language class throughout for consistency purposes.

**S2 Table. Mastery Goals- Intrinsic Goals**

| 1. In a class like this, I prefer course material that really challenges me so I can learn new things |
| --- |
| 1. In a class like this, I prefer course material that arouses my curiosity, even if it is difficult to learn |
| 1. The most satisfying thing for me in this course is trying to understand the content as thoroughly as possible |
| 1. When I have the opportunity in this class, I choose course assignments that I can learn from even if they don’t guarantee a good grade |

**S3 Table. Performance Goals- Extrinsic Goals**

| 1. Getting a good grade in this class is the most satisfying thing for me right now |
| --- |
| 1. The most important thing for me right now is improving my overall grade point average, so my main concern in this class is getting a good grade |
| 1. If I can, I want to get better grades in this class than most of the other students |
| 1. I want to do well in this class because it is important to show my ability to my family, friends, future employer, or others. |

**S4 Table. Task value**

| 1. I am very interested in the content area of this course |
| --- |
| 1. I like the subject matter of this course |
| 1. I think I will be able to use what I learn in this class in other courses |
| 1. It is important for me to learn the course material in this class |
| 1. I think the course material in this class is useful for me to learn |
| 1. Understanding the subject matter of this course is important for me |

**S5 Table. Metacognitive self-regulation**

| 1. I ask myself questions to make sure I know the material I have been studying |
| --- |
| 1. I work on practice exercises and answer end of chapter questions even when I don't have to. |
| 1. Even when study materials are dull and uninteresting, I keep working until I finish |
| 1. Before I begin studying, I think about the things I will need to do to learn |
| 1. When I'm reading, I stop once in a while and go over what I have read |
| 1. I work hard to get a good grade even when I don't like a class |

Note: Three items from the original scale were dropped from the analyses due to low latent factor loadings even after reverse-scoring due to negative item wordings.
